# Pesticides and Bladder Cancer: Mechanisms Leading to Anti-Cancer Drug Chemoresistance and New Chemosensitization Strategies

**DOI:** 10.3390/ijms241411395

**Published:** 2023-07-13

**Authors:** Christopher A. Lucchesi, Demitria M. Vasilatis, Saisamkalpa Mantrala, Thenappan Chandrasekar, Maria Mudryj, Paramita M. Ghosh

**Affiliations:** 1 VA Northern California Health Care System, Mather, CA 95655, USA; dvasilatis@ucdavis.edu (D.M.V.); mmudryj@ucdavis.edu (M.M.); 2 Department of Surgical & Radiological Sciences, School of Veterinary Medicine, University of California Davis, Davis, CA 95616, USA; 3 Department of Urological Surgery, School of Medicine, University of California Davis, Sacramento, CA 95817, USA; 4 Department of Medical Microbiology and Immunology, School of Medicine, University of California Davis, Davis, CA 95616, USA; 5 Department of Biochemistry and Molecular Medicine, School of Medicine, University of California Davis, Sacramento, CA 95817, USA

**Keywords:** bladder cancer, pesticide exposure, metastasis

## Abstract

Multiple risk factors have been associated with bladder cancer. This review focuses on pesticide exposure, as it is not currently known whether agricultural products have a direct or indirect effect on bladder cancer, despite recent reports demonstrating a strong correlation. While it is known that pesticide exposure is associated with an increased risk of bladder cancer in humans and dogs, the mechanism(s) by which specific pesticides cause bladder cancer initiation or progression is unknown. In this narrative review, we discuss what is currently known about pesticide exposure and the link to bladder cancer. This review highlights multiple pathways modulated by pesticide exposure with direct links to bladder cancer oncogenesis/metastasis (MMP-2, TGF-β, STAT3) and chemoresistance (drug efflux, DNA repair, and apoptosis resistance) and potential therapeutic tactics to counter these pesticide-induced affects.

## 1. Introduction

In the United States, approximately 82,290 people will be diagnosed with bladder cancer (BlCa) in 2023 [1]. California, which has the largest agricultural workforce, will see the greatest increase in BlCa cases of all the states (8.8% of all BlCa in USA) [2]. Of these patients, almost three quarters will have non-muscle invasive BlCa (NMIBC). Statistically, more than half of NMIBC will recur within 1 year after the first treatment and one third of cases will progress to muscle invasive BlCa (MIBC) [3]. Low-grade NMIBC is commonly managed with transurethral resection (TUR) with 5-year overall survival of 90% [4]. The 5-year survival for non-metastatic MIBC is 70%, which is in stark contrast to metastatic-MIBC, which has a precipitous decrease in survival to 38% when regional and 6% when distant metastases are present. These data ultimately highlight the necessity to better understand BlCa progression [5].

In the developed world, BlCa is the 10th and 11th most common cancer in men and women, respectively [6]. Multiple risk factors have been associated with BlCa; for example, tobacco users are nearly three times as likely to get BlCa [1]. The leading risk factors for BlCa include cigarette smoking and occupational exposures [7]. Multiple aromatic amine compounds, such as 4-aminobiphenyl, benzidine, 2-naphthylamine, and ortho-toluidine have been associated with BlCa prevalence in the occupational setting [8]. Additionally, the American Cancer Society also lists certain medications (e.g., pioglitazone, a drug used in the management of type 2 diabetes mellitus) and herbal supplements (mainly herbs from the *Aristolochia* family), arsenic in drinking water, and lack of fluids as risk factors for BlCa [9]. Non-hispanic Caucasians are about twice as likely to develop BlCa as African Americans (AA) and Hispanic individuals; however, AAs develop more advanced disease and have a worse prognosis [10,11]. The risk of BlCa also increases with age and is much more common in men than in women [12]. BlCa has been associated with an inflammatory/immune response to irritants [13]. Urinary infections, kidney and bladder stones, and other irritants, such as catheters left in the bladder for prolonged periods, have been linked to the development of squamous cell carcinoma (SCC) of the bladder [14]. Schistosomiasis, a parasite, has also been known to cause bladder SCC, mostly in Africa and the Middle East [15]. Birth defects, such as exstrophy, greatly increase the risk of BlCa (likely due to mucosal metaplasia), as do chemo- and radiotherapy for other cancers, such as prostate cancer, where the treatment affects the bladder [16,17,18,19].

Pesticides, which are extensively used in agricultural work, have also been linked to cancer in residents of rural areas who are exposed to agricultural products [20]. Multiple studies have demonstrated a strong correlation between pesticide exposure and increased incidence rate of BlCa in humans and other mammals, such as dogs, exposed to pesticides. For example, exposure to pesticides among agricultural workers (men and women) is correlated with increased risk of BlCa, as well as nonoccupational exposure to agricultural work [21,22]. Further, multiple studies from various countries showed increased BlCa incidence among farm workers exposed to pesticides [23,24,25].

It should be noted that agricultural populations have a lower prevalence of smoking than does the general population, supporting the hypothesis that the increased risk of BlCa in this group can likely be attributed to other causes [26,27]. Supporting this, two studies demonstrated a link between farming and BlCa amongst non-smoker workers [28,29]. Pesticides and their metabolites are expelled from the body via the urine, increasing the risk for pesticides which negatively affect the bladder [30]. Recent evidence suggests that pesticide exposure regulates the methylome, translatome and metabolome highlighting potential mechanisms by which pesticide exposure can promote oncogenesis. Despite this evidence, the mechanism by which pesticide exposure contributes to BlCa and oncogenesis is still poorly understood. In this review we consider the effects of pesticide exposure on oncogenesis and metastasis, chemoresistance, and potential therapeutic modalities for BlCa. We conclude that among farm workers and those living near farms, pesticide exposure may be a significant cause of BlCa development.

## 2. Structure of the Bladder

**Figure 1:**
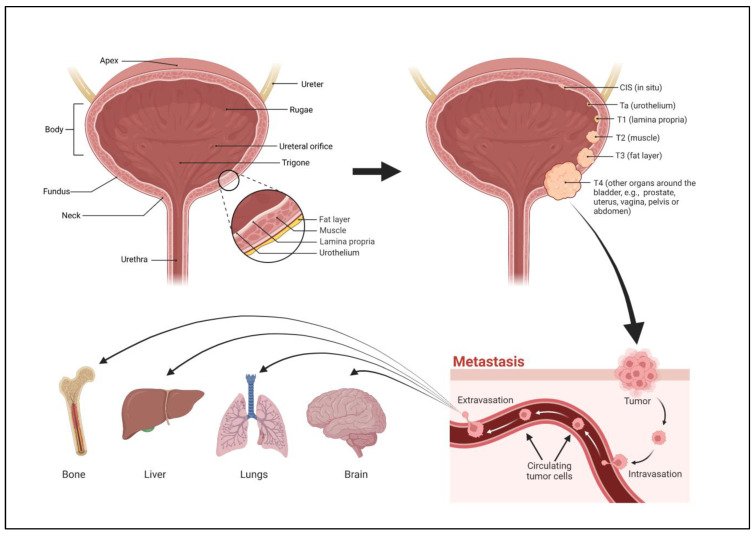
Visual schematic of the bladder, stages of bladder cancer, and mode of metastasis.

The bladder is a hollow organ located in the lower abdominal region. The bladder is contiguous with the ureters above, through which urine flows from the kidneys, and the urethra below, which dispels urine from the body. There are four parts to the bladder, the apex or dome, body, fundus, and neck (Figure 1). The apex is the anterosuperior part of the bladder that points towards the abdominal wall. The fundus, or base, is the posteroinferior part of the bladder. The fundus lies on the inferior aspect of the proximal wall while the apex lies on the anterior aspect of the wall, extending towards the pubic symphysis. The body of the bladder is the large area situated between the apex and the fundus. The neck of the bladder is the constricted part of the bladder that leads to the urethra [31]. The upper part of the bladder consists of the apex and body which are above the ureteric orifices. The lower part consists of the fundus, neck, and trigone. The trigone is an inverted triangular-shaped area of space that is made entirely of smooth muscle. Its superolateral angles are formed by the ureteric orifices. The neck lies at the base of the trigone and it is the most inferior point of the bladder [31].

The carefully coordinated action of the bladder is determined largely by its histology which facilitates its physiological function [32]. The innermost layer of the bladder is the transitional epithelium, also known as the urothelium (Figure 1). This lining is consistent throughout the ureters and urethra and is known for its elasticity. Because urine is stored in the bladder for extended periods of time, the urothelium functions to prevent diffusion of urinary components into the underlying tissues [33]. The next layer is the lamina propria (LP) which is composed of an extracellular matrix containing several types of cells, including fibroblasts, adipocytes, interstitial cells, and afferent and efferent nerve endings [34]. The LP, due to its components, allows the urothelium to send messages to the other layers of the bladder wall, contributing to the activation of the detrusor muscle which lies outside it. Together, the LP and urothelium form the mucosa of the bladder. When empty, the mucosal layer exists in a highly folded state, creating pleats of tissue known as rugae, which allows the bladder to expand when filled with urine. Beyond this is the muscularis, composed of interwoven smooth muscle fibers that are known as the detrusor smooth muscle (DSM). Contraction and relaxation of the DSM leads to micturition (urination) [35]. The bladder is highly innervated, and urination relies on the relaxation of the internal sphincter and contraction of the bladder walls. This expels the urine into the urethra, where excretion is dependent on the relaxation of the external sphincters which are voluntarily controlled.

## 3. Stages of Bladder Cancer

Urothelial carcinomas (UC) can be divided into two categories: NMIBC and MIBC. Approximately 75% of UC are NMIBC [36]. According to WHO classification systems, NMIBC are further divided into three stages: Carcinoma in situ (CIS), Ta (non-invasive papillary), and T1 [37]. These can be further sub-graded as either low or high-grade. Both Ta and CIS are considered non-invasive (Figure 1). Stage CIS is characterized by a flat lesion tumor which remains in the tissue layer from which it originates without spreading into the hollow space of the bladder. Ta, a papillary tumor, grows into the hollow area of the bladder with finger-like projections but does not spread to the connective tissue or muscle tissue of the bladder wall [38]. Cancers in the Ta and CIS stages account for 70% and 20% of all NMIBC, respectively [39]. Tumors in stage T1 spread into the connective tissue layer (lamina propria) under the lining layer of the bladder, but do not spread into the muscle layer. In general, NMIBC is characterized by high recurrence of tumors and a high survival rate after therapeutic mechanisms are employed [40].

MIBC, as the name indicates, indicates tumors that have invaded into the muscularis propria (DSM). This type of BlCa is divided into three distinct stages, T2, T3, and T4, with each stage becoming progressively worse and indicating a more serious prognosis. T2 is characterized by the spreading of the cancer into the inner layer (T2a) or outer layer (T2b) of the muscle of the bladder wall, but the cancer has yet to spread beyond the muscle layer into the fatty tissue layer that surrounds the bladder. Once it moves into the perivisceral tissue, the tumor can be characterized as T3a if it can be seen microscopically, or T3b if it can be visualized macroscopically during imaging and/or can be physically palpated by the physician. When the tumor has spread to the surrounding organs including the prostate and seminal vesicles in males, or the uterus and vagina in females, it is considered T4a. T4b is when the cancer has not only invaded the organs mentioned above but also the pelvic and abdominal wall [38]. Further, MIBCs can be grouped into basal and luminal subtypes—basal MIBCs are associated with shorter disease-specific and overall survival, while luminal MIBCs were enriched with FOXA1, GATA3, ERBB2 and ERBB3, and responded to neo-adjuvant chemotherapy [41].

## 4. Grades of Bladder Cancer

Grading of bladder cancer is differentiated by a pathologist based on histological diagnosis. Low-grade NMIBC is characterized by high recurrence rates, but rare propensity (10–15%) to progress into high-grade BlCa, invade, or metastasize [42]. High-grade NMIBC comprises the tumors that are characterized by a quick progression into invasive tumors, those of the MIBC category [43]. Ta often recurs after surgical resection, but rarely progress to high-grade T1 tumors or to MIBC (seen in <10% of cases). In contrast, CIS tumors progress in about 50% of cases to T1 and then to MIBC. About 80% of MIBC are thought to arise from CIS tumors.

Studies have shown that the ability for low-grade BlCa to progress into high-grade BlCa is likely due to acquired TP53 mutations, whereas *TP53* mutations are more frequent in high-grade BlCa, and *FGFR3* mutations are more frequent in low-grade tumors [44]. Almost all mortality from BlCa results from high-grade disease. It may be noted that the low vs. high grade designation is typically used only for transitional cell carcinoma. For squamous cell carcinoma or adenocarcinoma, a gradation system of well, moderate, or poorly differentiated cancers is used.

## 5. Types of Bladder Cancer

BlCa is a heterogeneous disease with multiple subtypes and variants. The most common histology of BlCa is urothelial carcinoma (UC) (also known as transitional cell carcinoma, TCC) that originates in the urothelial cells of the bladder. UC accounts for up to 90% of all BlCa diagnoses [45].

Certain variants of UC elucidated by the World Health Organization (WHO) are rare and hard to diagnose. UC with squamous differentiation (UCSD) is the most common histological variant, found in T1 tumors 8–15% of the time and 20–60% of the time in MIBC. It presents with a significantly higher pathologic stage than pure UC and is defined as high-grade [46]. UCSD is defined by the presence of clear-cut intercellular bridges, intracellular keratinization, or both [47]. As other works have reported, squamous differentiation is significantly correlated with multiple severe prognostic factors including progression to grade 3, lymphovascular invasion, advanced tumor stage, and lymph node metastasis [48]. UC with glandular differentiation (UCGD) is the second most common histological variant and is found in around 10% of all UC cases. This kind of differentiation is more often visualized as urothelial carcinoma with gland-like lumina [47]. Glandular differentiation can exist either in the form of pure adenocarcinoma (rarer and largely associated with schistosomiasis as well as bladder exstrophy) or UCGD [49].

Secondary to UC is squamous cell carcinoma (SCC) of the bladder. SCC accounts for the remaining 10% of BlCa cases and is more common in Africa where it is associated with the protozoan infection schistosomiasis [12,15]. SCC is largely characterized by keratin deposits [50]. It is of important clinical significance to distinguish SCC from UCSD as the latter is treated similarly to pure UC while the former is resistant to chemotherapy requiring radical cystectomy (RC) without neoadjuvant chemotherapy (NAC) [49]. Chronic irritation of the bladder due to bladder stones, multiple urinary tract infections, or long-term indwelling catheters can lead to SCC and can initiate metaplasia from transitional cells to squamous cells [51,52]. SCC has a significantly worse prognosis than UC [53]. Other, rarer forms of BlCa include adenocarcinoma which is only found in 0.5–2% of all cases and small cell carcinoma which is found in less than 1% of all BlCa cases [54]. Small cell carcinoma is poorly differentiated, and highly metastatic with a poorer prognosis than pure UC [55].

## 6. Pesticides

Pesticides, substances used to destroy insect and other organisms harmful to cultivated plants or animals, are an expansive group of diverse chemicals that have a substantial public health advantage via increasing food production yield; nevertheless, depending on the chemical and/or route of exposure, they may pose a significant health risk [56]. Detrimental exposure to pesticides can occur from occupational contact, agricultural and household use, and through unwashed food consumption [57]. Inappropriate pesticide handling and application practices substantially increase the risks of exposure, while unnecessary pesticide application exceeding the recommended rates can result in the accumulation of pesticide residue in the environment and in food products [58,59].

The US Environment Protection Agency (EPA) recognizes multiple types of pesticides, including insecticides, herbicides, rodenticides, and fungicides. However, other compounds may also fall in this category—such as disinfectants, attractants, plant defoliants, swimming pool treatments, and plant growth regulators. Diverse types of pesticides including organophosphorus, organochlorines, pyrethroids, and carbamates are commonly used in tropical and developing countries [60]. Studies have shown that while acute exposure to these compounds may result in poisoning, chronic exposure to small doses of the same compounds also has substantial health risks. The use of these agents has been shown to lead to DNA damage caused by biotransformation of the compounds, via oxidation and hydrolysis, thereby increasing the risk of cancer [61]. Further, when these agents are used in concentrated forms, their metabolites have been detected in quantifiable levels in multiple bodily fluids including blood, urine, saliva, and milk [62,63,64]. The health risks associated with pesticide exposure depend on the toxicity of the agent and the level of exposure [65]. Therefore, the length of exposure and concentration of the agent, which is absorbed into the body, greatly influence the intensity of detrimental effects of pesticide [66]. Populations living within areas with high pesticide use have an increased risk of developing multiple types of cancers [67], including non-Hodgkin’s lymphoma, multiple myeloma, soft-tissue sarcoma, lung sarcoma, pancreatic, skin, brain, stomach, liver, bladder, and gall bladder cancer [61]. Table 1 demonstrates commonly used pesticides that have been linked to bladder cancer, and Table 2 describes those that have been associated with other cancers. Beyond cancer, long-term pesticide exposure has also been associated with amplified abnormality of nerve conductions, neurotoxicity, psychiatric problems, cognitive defects including memory loss and learning impairment, and genetic damage [68,69,70,71].

## 7. Routes of Pesticide Exposure That May Promote Bladder Cancer

There are three routes of entry for pesticides into the human body including dermal (through skin and/or eyes), respiratory (inhalation), and oral (ingestion). For the general population, the primary route of exposure is through ingestion of treated foods and from exposure in residential areas. One possible mode of action potentiating BlCa risk by pesticide exposure is by the “urogenous contact” hypothesis which suggests that active carcinogens created via pesticide metabolites dissolved in urine can transform cells of the bladder epithelium [104,105]. For a more complete review on the pharmacokinetics and metabolism of pesticides please see [106].

Animal models are frequently used to evaluate and understand the routes of exposure in humans [107]. Dogs exposed to pesticides are at an increased risk of developing BlCa and may serve as a sentinel species for human exposure. In one study, dogs known to be exposed to lawn herbicides and insecticides, or herbicides alone, had a 7.2-fold increased risk of developing BlCa when compared to non-exposed dogs [76]. Additionally, the risk of developing BlCa was also found to be higher in dogs living near marshland sprayed with insecticides [108]. The most common herbicides that dogs were exposed to included phenoxy acids such as 2,4-dichlorophenoxyacetic acid (2,4-D), 2-(di fluoromethyl)-4-(2-methylpropyl)-6-(trifluoromethyl)pyridine-3,5-dicarbothioate (MCPP), 4-chloro-2-methyl phenoxy acetic acid (MCPA); benzoic acids such as 3,6-dichloro-2-methoxybenzoic acid (dicamba); and organophosphorus compounds such as *N*-(phosphonomethyl)glycine (glyphosate) [76]. Of these, several compounds have been detected in dog urine via LC/MS and are speculated to be the major cause of BlCa in these animals. These grass herbicides were not only found in the experimental group that had grass herbicide use at home, but in the control group which did not have grass herbicide use at home, which has been speculated to be due to exposure outside of the yard or due to herbicidal drift [109].

In support of the above statement, unlike humans, female dogs are more prone to BlCa when compared to male dogs at a ratio of 1.71–1.95:1 [110]. This may be due to increased urine retention in female dogs and subsequently increased exposure of the urinary epithelium to carcinogens. Female dogs do not display urinary marking behaviors as frequently as male dogs, causing them to retain urine for longer periods of time. In addition, increased body fat content of female dogs potentially aids in retaining more lipophilic environmental carcinogens. Collectively, both humans and companion animals exposed to pesticides show a strong correlation to BlCa initiation.

## 8. Role of Pesticides in Oncogenesis

Data from the Agricultural Health Study (AHS), a large prospective cohort study of pesticide applicators with detailed pesticide use data, was used to evaluate the association between several specific pesticides and bladder cancer risk [30]. Among herbicides, 2,4-D, bentazon, bromoxynil, chloramben, diclofop-methyl, imazaquin, and sethoxyzim, were identified to have significant risk, while among insecticides, dichlorodiphenyltrichloroethane (DDT) and heptachlor were found to impose significant health risks. None of the fumigants or fungicides had similar effects. 2,4-D is not genotoxic and showed no carcinogenesity in laboratory animals. However, it was found to have significant effects in humans [111]. It was one of the chemicals found in the urine of pet dogs exposed to lawn pesticides [109]. 4-chlorophenol, a metabolite of 2,4-D, has also been detected in the urine of both pet dogs and their owners [112]. None of the other herbicides named have been studied extensively in BlCa, and in general, their mechanism of action at the molecular level is not well understood.

Increased levels of various organochlorine and organophosphorus pesticides including α-hexachlorocyclohexane (α-HCH), β-HCH, γ-HCH, 2,4-DDT), 4,4-DDT, 2,4-dichlorodiphenyldichloroethylene (2,4-DDE), and 4,4-DDE were found in BlCa patient serum at higher levels than controls [113]. This was accompanied by a significant decrease in acetylcholinesterase (AChE) activity, and a significant increase in malondialdehyde (MDA) levels in serum of BlCa patients while arylesterase activity of paraoxonase-1 (ARE), and total antioxidant capacity (TAC) levels were marginally affected. Organophosphates are known AChE inhibitors and prevent breakdown of acetylcholine (ACh), a naturally occurring neurotransmitter, into acetic acid and choline. AChE terminates neuronal transmission and signaling between synapses to prevent ACh dispersal and receptor activation; therefore, a sudden and strong suppression of AChE, as caused by organophosphates, may result in cholinergic poisoning. On the other hand, increased MDA levels are a marker of oxidative stress and has been shown to indicate antioxidant status in cancer patients. An increase in MDA levels is frequently seen in patients exposed to pesticides [114].

Pesticides have been shown to drive mitochondrial dysfunction which potentiates oncogenesis by producing a metabolic shift to anaerobic conditions which may also include mutations in mitochondrial genes [115]. DDT causes defects in succinate dehydrogenase (complex II), as well as other mitochondrial enzymes, which has been associated with both familial and sporadic forms of cancer [116,117]. Through its interruption of mitochondrial OxPhos, DDT induces a large amount of oxidative stress. This imbalance of oxidative stress can drive oncogenesis partially through its activation of various transcription factors including NF-κB, AP-1, HIF-1α, PPAR-γ, β-catenin/Wnt, and Nrf2. Aberrant activation of these transcription factors leads to the expression of numerous genes encoding for growth factors, inflammatory cytokines, chemokines, cell cycle regulatory proteins, and anti-inflammatory proteins [118]. Organophosphate exposure was correlated with acquisition of K-Ras mutations in BlCa patients [119], and K-Ras mutations have been shown to drive bladder oncogenesis [120]. Genetic mutations in FGFR3, RB1, HRAS, TP53, TSC1 and other genes on chromosomes 9 and 22 are also known to drive bladder oncogenesis. Many chemicals including organophosphates were shown to induce mutations in these genes, which may account for an increased incidence of BlCa in these individuals [121].

## 9. Role of Pesticides in EMT and Metastasis

Metastasis is the development of distant secondary tumors away from the primary cancer [122]. It is responsible for the largest number of cancer-related deaths and is the main cause of treatment failure and mortality in BlCa patients [123]. For metastases to occur, cancer cells must leave their primary site, circulate in the bloodstream or lymphovascular system, acclimatize to a new cellular surrounding at the secondary site by adapting to supportive niches (Figure 1), and escape the immune system [124,125,126]. The various steps of metastasis all require cytoskeletal rearrangements, coupled with secretion of extracellular matrix metalloproteinases (MMP) [127,128,129,130]. In recent years, it has been shown that pesticide exposure can modulate many of these metastatic traits. Not all the pesticides listed above have been associated with bladder cancer, but we are listing them here as they are all detected in the urine and have the potential to affect BlCa.

Epithelial mesenchymal transition (EMT) has been shown to be a key driver of metastasis [131] and is of great therapeutic interest [132]. EMT is a reversible change that reduces intercellular adhesion and causes epithelial polarization [133]. It endows cancer cells with migratory and invasive properties, stimulates stem cell properties, while also preventing apoptosis and senescence, and contributes to immunosuppression, which ultimately promotes metastasis [134]. EMT is characterized by loss of E-cadherin and increased expression of MMP-9 associated with a poor clinical outcome in BlCa patients. Multiple pesticides were found to enhance EMT through alterations in EMT related genes and pathways as described below.

E-cadherin is a cell–cell adhesion protein that regulates epithelial cell junctions and is frequently lost in advanced cancers. The extracellular region of E-cadherin extends to the cadherins of adjacent cells while the intracellular region binds to β-catenin at the C-terminal. Release of β-catenin from E-cadherin results in β-catenin binding to transcription factors and gene transcription. The organophosphate insecticide malathion, developed to prevent disease by controlling mosquitos, was found to induce alterations in the actin cytoskeleton and decrease the expression of adhesion molecules E-cadherin and β-catenin in breast cancer lines [88]. While high doses of malathion were highly toxic, low dose exposure to this compound resulted in loss of E-cadherin accompanied by an increase in the expression of Rho and Rac1 GTPases, which promotes actin cytoskeleton rearrangements migration and invasion. While similar effects have not been reported in BlCa, malathion has been detected in the urine and is thought to accumulate in the bladder even due to dermal exposure [135].

It has been demonstrated that enhanced transforming growth factor-β (TGF-β) signaling promotes EMT. In the past, arsenical herbicides were extensively used in the US and elsewhere. Dimethylarsinic acid (DMA), the major urinary metabolite of inorganic arsenic, is a urinary bladder carcinogen that increased TGF-β secretion in rat urine [136]. A separate study found that DMA induced EMT through several mechanisms including upregulation of HER2 that is also known to increase TGF-β [137]. Paraquat poisoning is also known to increase TGF-β levels in alveolar type II (AT II) cells [138] but also in zebrafish [139].

The effect of pesticides on TGF-β and resulting EMT is not confined to BlCa alone. Paraquat exposure induced EMT-like cellular response resulting in fibrogenesis in human pulmonary epithelial cells [79]. Occupational exposure to pesticides significantly upregulated TGF-β1 in breast cancer cells [140], and the pesticide endosulfan was shown to modulate the TGF-β/Smad signaling pathway leading to cell proliferation and EMT-like characteristics in human renal mesangial cells [85]. The hepatocyte growth factor (HGF)/c-Met signaling pathway is strongly associated with BlCa oncogenesis and patient prognosis [141], and c-Met activation was also found to upregulate TGF-β signaling [142]. Taken together, these studies suggest that TGF-β-induced EMT may be an important contributor of metastasis in BlCa upon pesticide exposure. The HGF/c-Met/TGF-β pathway, though, is relatively easy to neutralize. Multiple HGF/c-Met neutralizing antibodies and small molecule inhibitors have been developed which are currently in preclinical and clinical trials [143]. Further, the TGF-β pathway has been pharmacologically targeted using a multitude of approaches including small molecule inhibitors, TGF-β-directed chimeric monoclonal antibodies, ligand traps, and vaccines [144]. Organophosphorus pesticide exposure enhanced the protein expression of EMT associated proteins vimentin, ax1, and slug [145], while chlorpyrifos (CPF), one of the most frequently used pesticides around the world, increased MMP2 and vimentin expression driving breast cancer invasion [90].

**Figure 2:**
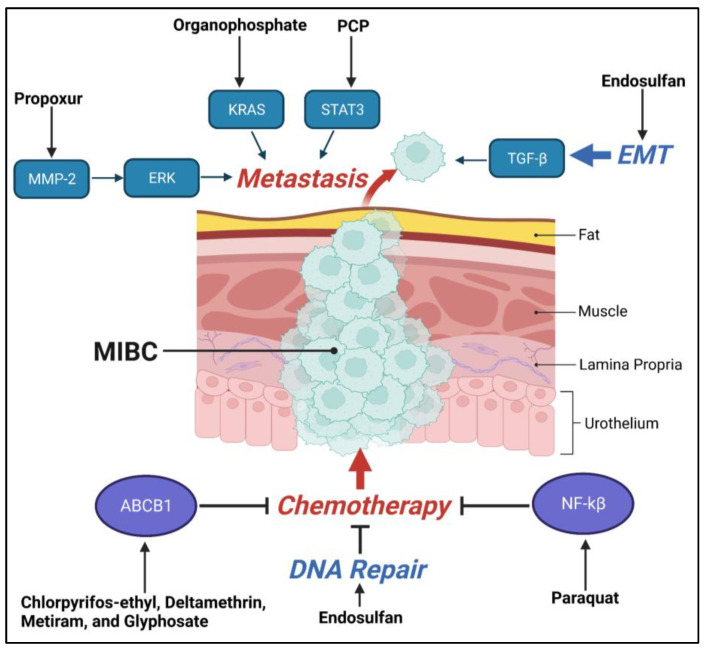
Visual schematic of the effects of pesticide exposure on key pathways contributing to tumor metastasis.

Another driver of oncogenesis and metastasis is Signal transducer and activator of transcription 3 (STAT3), which is constitutively activated in many cancers, and has been shown to be activated via pesticide exposure [92]. STAT3 can regulate cellular proliferation, invasion, migration, and angiogenesis that are all critical for cancer metastasis, again highlighting the mechanism by which pesticide exposure is capable of modulating metastasis [146]. A significant marker for BlCa cell proliferation is an increase in phosphorylated STAT3 (pSTAT3), which concurrently increased with disease progression [147]. STAT3 phosphorylation is induced by the cytokine IL-6, one of the main activators that allows STAT3 to bind to vascular endothelial growth factor (VEGF) and consequently promote tumor angiogenesis [147]. Studies utilizing human liver carcinoma cells (HepG2) showed that treatment with the pesticide pentachlorophenol for 24 h resulted in a significant induction of cytokines and chemokines including IL-6 [93]. IL-6 has been found to be overexpressed in BlCa specimens in comparison to non-malignant tissues at both the mRNA and protein levels. This increase directly correlated with a higher clinical stage, a higher recurrence rate after curative treatment, and reduced survival rate [147]. Therefore, a connection may be established between chronic pesticide exposure, increases in levels of IL-6, and progression of BlCa. Collectively, these data support the notion that pesticide exposure can modulate many processes contributing to metastasis (Figure 2).

## 10. Chemoresistance Due to Pesticide Exposure

Low grade NMIBC is commonly managed with transurethral resection (TUR) and has a 5-year overall survival reaching 90%. In contrast, the 5-year survival of patients with regional and distant metastasis is 38% and 6%, respectively. The standard of care treatment for MIBC involves neoadjuvant platinum-based chemotherapy (NAC) followed by radical cystectomy (RC [148]. This approach can improve survival in patients with MIBC compared with RC alone; however, only ~50% of MIBC patients respond to chemotherapy [149]. Many patients are also treated with adjuvant chemotherapy after RC. Patients that cannot undergo cystectomy have other nonequivalent options—they may undergo fulguration following transurethral resection (TUR) to destroy abnormal growths or tissues [150], while others may undergo radiation therapy, immunotherapy, or chemotherapy. Most common chemotherapeutic treatments for MIBC involve platinum-based compounds—cisplatin and carboplatin in combination with methotrexate, vinblastine, doxorubicin (MVAC), and gemcitabine (GC [151].

Chemoresistance is a major challenge in the treatment of BlCa [151]. There are numerous mechanisms associated with chemoresistance including (1) drug efflux where cancer cells pump chemotherapy drugs out of the cell using efflux pumps, such as ATP-binding cassette (ABC) transporters, which reduces the concentration of the drug inside the cell making it less effective [152,153]; (2) enhanced DNA repair capacity (DRC) allows cancer cells to limit the toxicity of DNA damaging chemotherapies [154,155]; and (3) intrinsic or acquired resistance allowing cancer cells to escape programmed cell death [156,157].

DNA repair pathways are activated in mammalian cells when they are exposed to endogenous or exogenous DNA-damaging agents to maintain genetic stability. A few of the frontline anti-cancer therapies, ionizing radiation, and chemotherapeutic agents, induce apoptosis by directly or indirectly causing DNA damage [155]. Therefore, alterations to DNA damage response may contribute to chemoresistance. Cells constantly repair DNA damage caused by extrinsic and intrinsic insults. To repair the harmful effects of these double stranded breaks (DSBs), our cells have evolved specialized machinery to sense and repair DNA damage. The two main mechanisms to repair DSBs are non-homologous end joining (NHEJ) and homologous recombination (HR). Interestingly, recent reports have identified an error-prone mechanism of end joining which is referred to as alt-NHEJ. This error-prone repair pathway significantly increases the mutational rate which drives disease progression and drug resistance [158,159]. This pathway has also been observed in highly mutagenic BlCa tumors contributing to their genomic instability [160]. Exposure to the organochlorine pesticide endosulfan was shown to lead to a significant increase in alt-NHEJ pathways leading to genomic instability in CML and ALL-derived cell lines and in BALB/C mice and Wistar rats [161]. Endosulfan is an organochlorine insecticide that is gradually being phased out due to toxicity. A study of 140 BlCa patients and 206 controls in the Canary Islands, however, did not find any association between endosulfan exposure and increased BlCa risk [162]. Nevertheless, oxidative stress induced DNA damage, as determined by an increase in 8-Hydroxyguanosine (8-OHdG), derived from guanosine by oxidative phosphorylation, was observed in sodium arsenite and DMA exposed urinary bladder damage in male rats as well as T-24 human BlCa cells [163].

Cancer cell evasion of apoptosis is another mechanism that promotes carcinogenesis, tumor progression and chemoresistance. Cancer cells have been shown to acquire apoptosis-resistance by modulating multiple pro-survival factors including NF-κB, and Bcl-2 family of proteins [164]. NF-κB has been shown to promote cell survival through induction of target genes which are inhibitory components of the apoptotic machinery. Therefore, cancers may use NF-κB to achieve resistance to anticancer drugs, radiation, and death cytokines [165]. Activated NF-κB functions as a transcription factor which modulates the gene expression of targets that prevent apoptosis. Functionally, NF-κB modulates pro-survival pathways through several antiapoptotic proteins including FLIP, Bcl-XL, A1/Bfl-1, IAP’s, XIAP, Survivin, TRAF1, and TRAF2 [166,167,168]. Therefore, it is unsurprising that aberrant expression of NF-κB has been shown to promote tumor progression and acquired chemoresistance [169]. Multiple studies have shown that NF-κB is significantly activated upon pesticide exposure [170,171,172] and is a regulator of transcription of EMT-related genes leading to enhanced metastasis [173,174,175], including BlCa [176]. Therefore, it is alarming that pesticide exposure activates NF-κB [172]. Paraquat was shown to increase NF-kB levels in the swim bladder of zebrafish [139]. Diallyl trisulfide (DATS), a component of garlic essential oil, is an effective insecticide for controlling aphids, found on most plants in yards and gardens. DATS reversed tobacco smoke-induced NF-κB pathway activation, EMT, and the acquisition of cancer stem cell-like properties in bladder tissues [177]. NF-κB and its pathways have been targeted using multiple biologics, macro- and small-molecule approaches [178].

Another pro-survival factor that is upregulated in BlCa is Bcl-2, associated with BlCa clinical stages and lymph node involvement [179], which inhibited apoptosis induced by the chemotherapeutic cisplatin [180]. One probable cause of Bcl-2 overexpression in BlCa is increased expression of NF-κB [181]. Additionally, it has been shown that the translocation of Bcl-2 gene near the immunoglobulin heavy chain gene locus (IGH) leads to overexpression of Bcl-2 [182]. Two studies have demonstrated that pesticide exposure leads to a much higher risk of developing the Bcl2-IGH translocation resulting in various diseases in human patients [183,184]. DATS induces apoptosis in T24 human bladder cancer cells by suppressing Bcl-2 expression [185]. DMA promoted BlCa in male Wistar rats partly by increasing Bcl-2 levels [186]. Collectively, this evidence strongly supports that pesticide exposure can drive acquired chemoresistance and is a plausible cause for poor patient response to BlCa treatment.

The ATP binding cassette subfamily B member 1 (ABCB1) (also called multi drug resistance 1—MDR1) belongs to the ABC transporter family which includes 48 members in humans. ABCB1 is one of the most characterized transporter proteins that is shown to be overexpressed in many cancers [187]. ABCB1 functions as an efflux pump which can exclude chemotherapeutic and other clinical agents from the cells [188]. Notably, multiple mutations (R433S, C43S, A989T and C1047S) and polymorphisms in ABCB1 have been associated with increased chemoresistance in human embryonic kidney and HeLa cells [189,190,191]. Markedly, pesticide exposure was correlated to increased ABCB1 mutations which may lead to chemoresistance and oncogenesis [192,193]. Interestingly, fruit-farmers harboring ABCB1-T129C polymorphism, when exposed to pesticides, had significantly higher DNA damage as compared to wild-type (WT) individuals. This increased DNA damage can result in the accumulation of deleterious DNA mutations which may contribute to oncogenesis [192]. Further compounding these finds, chronic exposure of glioblastoma cells to a low-dose mixture of pesticides (chlorpyrifos-ethyl, deltamethrin, metiram, and glyphosate) lead to the over expression of ABCB1, as well as BRCP/ABCG2 and glutathione-S-transferase (GST)/M1-type cellular detoxification function, all of which may lead to chemoresistance [80]. Resistance to cisplatin in bladder cancer has long been associated with the activation of ABCB1 and may represent an additional mechanism by which pesticides induce resistance to chemotherapy in exposed individuals [194]. This study demonstrated that the exposure to low-dose mixture of pesticide caused acquired multidrug chemoresistance, and further, this resistance was transferable to subsequent cell generations.

## 11. Pesticide Effects on Other Therapies for Bladder Cancer

There are currently other types of treatment for BlCa when chemotherapy fails. Erdafitinib, a small molecule inhibitor of fibroblast growth factor receptor (FGFR), is active in platinum refractory BlCa expressing FGFR3 mutations and FGFR2/3 fusions [195]. Antibody drug conjugates (ADCs) enfortumab vedotin, a nectin-4-directed antibody and microtubule inhibitor monomethyl auristatin E (MMAE) conjugate and sacituzumab govitecan, an anti-Trop-2-antibody conjugated to a topoisomerase inhibitor, also demonstrated activity in BlCa in vivo [196,197]. RC48-ADC (disitamab vedotin), conjugating the anti-HER2 antibody hertuzumab to MMAE, had activity in BlCa patients with HER2-amplified or mutated failing chemotherapy [198]. As increased HER2 expression has been found to be correlated with pesticide exposure, this may prove to be an effective therapeutic approach [199]. HER2 is a member of the epidermal growth factor receptor (EGFR) family and class I evidence revealed that DMA elevated EGFR activation in rat urothelial cells, and that the EGFR inhibitor gefitinib blocked DMA-induced cell proliferation in vitro as well as in the rat urothelium [200].

Cancer cells must also invade the immune system. A key modulator of immune invasion in many cancers, including BlCa, is programmed cell death protein 1 (PD-1) and its ligand PD-L1 [201]. Exposure to the pesticide Diuron, coupled with AKT overexpression, was found to be correlated with increased PD-L1 expression [140,202,203]. Recently, PD-1/PD-L1 inhibitors have been shown to be effective for multiple cancers, including balder cancer [202,203]. Interestingly, high PD-L1 expression, which is found in bladder cancer, has been shown to inhibit cis-platinum based chemotherapy response, which can be sensitized via metformin-modified chitosan [204]. In addition, it has been shown that pesticides can affect multiple immune cells and have been suggested as immunotoxic, leading to another mechanism for cancer cell immune evasion [205]. Diuron (3-(3,4-dichlorophenyl)-1,1-dimethylurea) is a substituted urea herbicide that induces rat urinary bladder urothelial tumors at high dietary levels (2500 ppm) [206]. Transcriptional profiling of diuron-induced toxicity on the urinary bladder of male Wistar rats demonstrated alterations in the CTLA4 checkpoint signaling after 20 weeks of exposure.

Propoxur exposure increased MMP-2 expression and invasion of cancer cells through the activation of the mitogen activated protein kinase (MAPK) member extracellular related kinase (ERK)/nuclear factor erythroid 2–related factor 2 (Nrf2) signaling in breast cancer [89]. It is a carbamate insecticide which induced urinary bladder cancer in Wistar rats when fed at 5000 ppm in the Altromin 1321 diet [207]. MMP-2 is one of the major collagenases in the MMP family which has been significantly correlated with BlCa progression through tumor invasion [208]. While previous MMP inhibitors had limited success and increased toxicities in the clinic, there is a new generation of MMP inhibitors currently in clinical trials which may prove useful in bladder cancer which has been shown to have high expression of multiple MMP family members [209]. Further, ERK1/2 predicts poor prognosis in BlCa, so the ERK1/2 inhibitor ulixertinib—which has shown promise in clinical trials in patients with advanced gastrointestinal malignancies—may also prove to be an effective therapeutic approach for BlCa [210,211].

## 12. Conclusions

While there is still limited direct information tying pesticide exposure to BlCa, the information that is known clearly demonstrates the effects of pesticide exposure may play a key role in the BlCa oncogenesis, progression, and chemoresistance. Further supporting this association, a bibliometric study of the past 10 years (2011–2021) reported that there is a significant rise in papers discussing the association between pesticide exposure and cancers, with BlCa being one of the top cancers cited [21]. Herein, this review highlighted the accumulating evidence which demonstrates a strong correlation of pesticide exposure to BlCa, not only in humans, but dogs alike. Pesticides were found to modulate key pathways involved in oncogenesis and metastasis, including MMP-2, STAT3, EMT, and TGF-β pathways. Beyond driving tumor progression, pesticide exposure was found to modulate various aspects of acquired chemoresistance such as drug efflux, enhanced DNA repair capacity, and acquired apoptosis resistance, all of which promote chemoresistance. As the risk of bladder cancer is correlated to pesticide exposure, it is paramount that we better understand these risks so that we can better protect those that are in direct contact with, and live in close proximity to, pesticides. Future studies are needed to research the risks associated with pesticide exposure as well as identify key pathways which may be targeted to bring novel therapeutics to the clinic for BlCa patients.

## Figures and Tables

**Table 1:** List of pesticides associated with bladder cancer and their structures.

Pesticide	Class	Structure	Potential Mode of Action	Reference
DDT	Insecticide	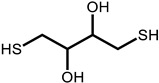	Oxidative stress, Xenoestrogen	[30,72,73]
Imazethapyr	Herbicide	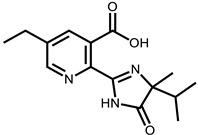	DNA adducts	[30,74,75]
2,4-Dichlorophenoxyacetic acid	Herbicide	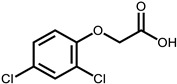	Oxidative stress	[30,76,77]
Bentazon	Herbicide	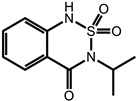	Mechanisms unknown	[30]
Bromoxynil	Herbicide	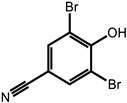	Mechanisms unknown	[30]
Chloramben	Herbicide	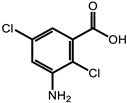	Mechanisms unknown	[30]
Diclofop-methyl	Herbicide	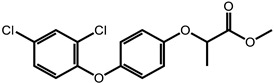	Genotoxic	[30,78]
Imazaquin	Herbicide	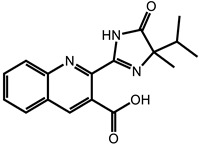	Mechanisms unknown	[30]
Heptachlor	Insecticide	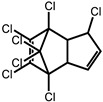	Mechanisms unknown	[30]

**Table 2:** List of pesticides and the cancers they are associated with.

Pesticide	Class	Cancer	Reference
Paraquat dichloride	Herbicide	Thyroid and Lung	[20,79]
Oxyfluorfen	Herbicide	Thyroid Cancer	[20]
Glyphosate	Herbicide	Thyroid Cancer, Glioblastoma	[20,80]
DDT	Insecticide	NHL, Prostate	[30,72,73]
Lindane	Insecticide	NHL, Prostate	[26,72]
Permethrin	Insecticide	NHL	[73]
Diazinon	Insecticide	NHL, Lung	[73,81]
Terbufos	Insecticide and Nematicide	NHL	[73]
Captafol	Fungicide	NHL	[82]
Simazine	Herbicide	Prostate	[72]
(Dithio/Thio)-carbamates	Fungicide	CNS Cancers	[83,84]
Imazethapyr	Herbicide	Colon	[30,74]
Cyhalothrin	Insecticide	DNA damage inducing	[60]
Endosulfan	Insecticide and Acaricide	Renal cancer, DNA damage inducing	[60,85]
Deltamethrin	Insecticide	Glioblastoma, DNA damage inducing	[60,80]
Polychlorinated biphenyls	Insecticide	Breast Cancer	[86]
Acetochlor	Herbicide	Lung Cancer	[87]
Malathion	Insecticide	Breast Cancer	[88]
Propoxur	Insecticide	Breast Cancer	[89]
Chlorpyrifos	Insecticide and Acaracide	Breast Cancer, Glioblastoma, Lung	[80,90,91]
Atrazine	Herbicide	Prostate Cancer	[92]
Pentachlorophenol	Termiticide, Fungicide, Herbicide, Molluscicide, Disinfectant	Lung, Liver	[93]
Metiram	Fungicide	Glioblastoma	[80]
EPTC	Herbicide	Pancreas, Colon	[94,95]
Dicamba	Herbicide	Lung, colon	[96]
Dieldrin	Insecticide	Lung	[97]
Metolachlor	Herbicide	Lung	[96]
Pendimethalin	Herbicide	Lung, Colon	[98]
Trifluralin	Herbicide	Colon	[99]
Chlordane	Insecticide (termiticide)	Colon, Leukemia, Prostate	[97,100]
Toxaphene	Insecticide	Colon, Melanoma	[91,97]
Fonofos	Insecticide and selective acaricide	Leukemia	[101]
Alachlor	Herbicide	Lymphoma	[102]
Aldicarb	Insecticide	Colon	[91]
Parathion	Insecticide and Miticide	Melanoma	[103]
